# Signal Peptide-Binding Drug as a Selective Inhibitor of Co-Translational Protein Translocation

**DOI:** 10.1371/journal.pbio.1002011

**Published:** 2014-12-02

**Authors:** Kurt Vermeire, Thomas W. Bell, Victor Van Puyenbroeck, Anne Giraut, Sam Noppen, Sandra Liekens, Dominique Schols, Enno Hartmann, Kai-Uwe Kalies, Mark Marsh

**Affiliations:** 1 KU Leuven – University of Leuven, Department of Microbiology and Immunology, Virology and Chemotherapy, Rega Institute for Medical Research, Leuven, Belgium; 2 Institute of Biology, CSCM, University of Lübeck, Lübeck, Germany; 3 MRC-Laboratory for Molecular Cell Biology, University College London, London, United Kingdom; 4 Department of Chemistry, University of Nevada, Reno, Nevada, United States of America; Medical Research Council, United Kingdom

## Abstract

In eukaryotic cells, surface expression of most type I transmembrane proteins requires translation and simultaneous insertion of the precursor protein into the endoplasmic reticulum (ER) membrane for subsequent routing to the cell surface. This co-translational translocation pathway is initiated when a hydrophobic N-terminal signal peptide (SP) on the nascent protein emerges from the ribosome, binds the cytosolic signal recognition particle (SRP), and targets the ribosome-nascent chain complex to the Sec61 translocon, a universally conserved protein-conducting channel in the ER-membrane. Despite their common function in Sec61 targeting and ER translocation, SPs have diverse but unique primary sequences. Thus, drugs that recognise SPs could be exploited to inhibit translocation of specific proteins into the ER. Here, through flow cytometric analysis the small-molecule macrocycle cyclotriazadisulfonamide (CADA) is identified as a highly selective human CD4 (hCD4) down-modulator. We show that CADA inhibits CD4 biogenesis and that this is due to its ability to inhibit co-translational translocation of CD4 into the lumen of the ER, both in cells as in a cell-free in vitro translation/translocation system. The activity of CADA maps to the cleavable N-terminal SP of hCD4. Moreover, through surface plasmon resonance analysis we were able to show direct binding of CADA to the SP of hCD4 and identify this SP as the target of our drug. Furthermore, CADA locks the SP in the translocon during a post-targeting step, possibly in a folded state, and prevents the translocation of the associated protein into the ER lumen. Instead, the precursor protein is routed to the cytosol for degradation. These findings demonstrate that a synthetic, cell-permeable small-molecule can be developed as a SP-binding drug to selectively inhibit protein translocation and to reversibly regulate the expression of specific target proteins.

## Abstract 2

A small chemical drug CADA specifically binds to the signal peptide of the membrane pre-protein CD4, disturbing its synthesis, impeding the routing to and expression on the cell surface.

## Author Summary

All cells are highly crowded with proteins that, once synthesized, have to reach their proper subcellular location in order to maintain the cellular homeostasis. Approximately 30% of the proteome needs to be sorted from the cytosol and inserted into, or transported through, biological membranes. For proteins sorted via the secretory pathway, an important step is the translocation into a cellular compartment called the endoplasmic reticulum (ER). The cell uses an elegant way to discriminate proteins that need to be translocated into the ER from those that have to reside in the cytosol by scanning for the presence of an N-terminal ER-entry tag. Although these tags, called signal peptides, have a common structure, they each contain a unique hydrophobic peptide sequence. In this work, we describe how a small chemical drug, CADA, can bind to one specific signal peptide present in the human CD4 pre-protein. We show that by influencing the signal peptide orientation in the translocation channel located in the ER membrane, CADA prevents CD4 translocation into the ER lumen. As a consequence, the CD4 protein is not properly synthesized and routed to the cell surface, resulting in a clear reduction in the amount of surface CD4, a membrane protein found on immune cells, and implicated in HIV-infection and other diseases. We believe that other drugs can be designed to selectively regulate, in a similar way, ER translocation of specific target proteins.

## Introduction

CD4 is a type I integral membrane glycoprotein that is expressed on the surface of thymocytes, T-helper lymphocytes, and cells of the macrophage/monocyte lineage [1]. It plays a central role in immune responses but also represents an obligatory component of the cellular receptor complex for HIV [2],[3]. Several reports demonstrate that down-modulation of surface CD4 protects cells from HIV infection [4]–[8]. In addition, natural CD4 down-modulation by memory CD4^+^ T cells *in vivo* protects African green monkeys from developing AIDS after infection with simian immunodeficiency virus (SIV), while maintaining the immunological functions normally attributed to CD4^+^ T cells [9]. Reduction in surface CD4 can be elicited by several factors that interfere with its translation or intracellular trafficking (reviewed in [10]). Phorbol esters are known to induce CD4 endocytosis through serine phosphorylation of the cytoplasmic tail of CD4 [11]. The concerted action of the three HIV-1 proteins Nef, Env, and Vpu results in a complete removal of CD4 from the surface of HIV infected cells through (i) enhanced routing of CD4 to the endoplasmic reticulum (ER) degradation pathway [12],[13] and (ii) activated endocytosis and lysosomal degradation [14],[15].

Surface expression of type I transmembrane (TM) proteins, such as CD4 receptors, requires translation of precursor proteins and their insertion into the ER membrane for subsequent routing to the cell surface. This co-translational translocation pathway begins when a hydrophobic N-terminal signal peptide (SP) on the nascent protein emerges from the ribosome and is recognized by the signal recognition particle (SRP). This complex of ribosome, nascent chain, and SRP is then targeted to the ER membrane via the interaction between SRP and its membrane receptor. Subsequently, the ribosome tightly binds to the Sec61 complex in the ER-membrane, a protein-conducting channel composed of the membrane proteins Sec61α, Sec61β, and Sec61γ. Finally, the ribosome continues the translation and the elongating polypeptide chain moves directly from the ribosome exit tunnel into the associated membrane channel. When the TM domain within the nascent polypeptide chain reaches the Sec61 complex, the channel opens laterally and the membrane anchor is released into the lipid bilayer (reviewed in [16],[17]). Simultaneously with the translocation of the polypeptide chain, cleavage of the signal sequence occurs at the luminal side of the ER together with other possible modifications such as N-glycosylation and proper folding of the polypeptide.

A screen for anti-HIV drugs led to the identification of CADA, a cyclotriazadisulfonamide with broad spectrum antiviral activity against laboratory strains and clinical isolates of HIV-1, as well as HIV-2 and SIV [18],[19]. The anti-HIV activity of CADA and its analogues correlated with their ability to down-modulate cell surface CD4 expression [8]. In the present study, we focused on the mechanism of action and molecular target of CADA. We demonstrate that CADA inhibits CD4 biogenesis during the early translational steps. More specifically, we show that (i) CADA selectively binds to the SP of human CD4 (hCD4), (ii) CADA prevents the growing CD4 polypeptide from entering the lumen of the ER, (iii) the SP of hCD4 is first inserted into the translocon channel with its N-terminus facing the lumen of the ER (N_lum_/C_cyt_) before an obligate inversion into a N_cyt_/C_lum_ topology takes place, and (iv) CADA locks the SP of hCD4 in an intermediate position during inversion and prevents further translocation of the polypeptide chain into the ER lumen.

## Results

### CADA Selectively Down-modulates Human CD4

**Figure 1:**
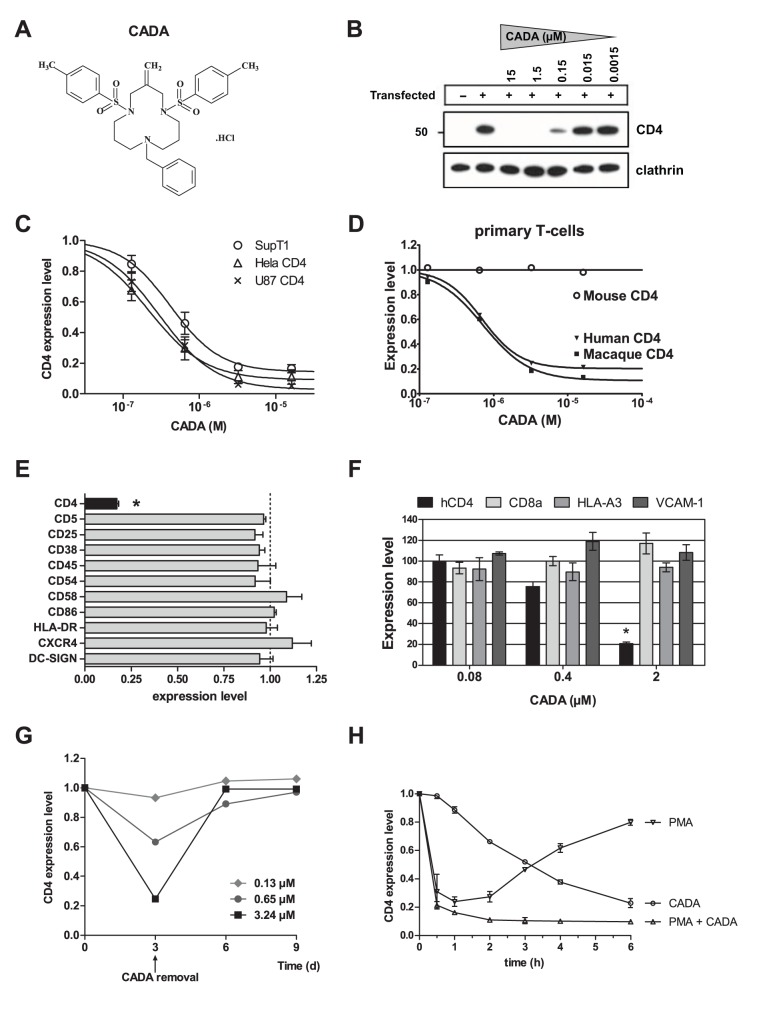
CADA selectively and reversibly down-modulates human CD4 in a dose-dependent way. (A) Structure of CADA, molecular weight (MW) = 618. (B) U87 cells stably expressing hCD4 were treated with a serial 1∶10 dilution of CADA. After 24 h, cell lysates were analyzed by immunoblotting with anti-CD4 or anti-clathrin antibodies. A non-transfected control is included (first lane). Molecular mass is in kDa. (C) Dose-response curve showing the effect of CADA on hCD4 expression in the human T-lymphoid cell line SupT1 expressing CD4 naturally (circles; IC_50_ is 0.55 µM), in the stably transfected human U87 glioblastoma (crosses, IC_50_ is 0.32 µM) and Hela epithelial cervical cancer cells (triangles, IC_50_ is 0.27 µM). Surface CD4 expression levels were normalized to non-treated controls as determined by flow cytometry (*n*≥4). (D) Dose-response curve showing the selective effect of CADA on primate CD4. Primary T-cells isolated from the blood of humans, macaques, or mice were stimulated with 2 µg/ml phytohaemagglutinin (PHA) and treated with CADA for 3 days. Next, cells were stained for surface CD4 and analyzed by flow cytometry. Data represent mean values from two different donors. (E) Human T-lymphoid MT-4 cells were treated with control medium or 3.2 µM CADA. After 24 h, cell surface proteins were quantified by flow cytometry. DC-SIGN expression was determined in stably DC-SIGN-transfected CEM cells. Protein expression levels were normalized to non-treated controls (three independent experiments of 10,000 analyzed cells each). *p<0.01. (F) Flow cytometry analysis of surface proteins in transfected HEK293T cells treated with CADA for 48 h. Protein expression levels were normalized to non-treated controls (three independent experiments). *p<0.01. (G) CD4 down-modulation by CADA is reversible. Primary human T-cells isolated from the blood of healthy donors were stimulated with 2 µg/ml PHA and treated with CADA for 3 days. Next, cells were washed and given control medium for another 6 days. Cells were harvested at specified time points and analyzed for surface CD4 expression by flow cytometry. Data represent mean values from two different donors. (H) Kinetics of CD4 down-modulation showing a slow but steady removal of surface CD4 with CADA, and a fast but transient decrease in CD4 with PMA. Graph represents flow cytometric surface CD4 analysis of CHO cells stably expressing hCD4 either treated with 16 µM CADA or 0.16 µM PMA, or treated simultaneously with CADA and PMA. Surface CD4 expression levels were normalized to non-treated controls (*n*≥2).

To evaluate the effect of CADA on CD4 expression, different cells were treated with the compound under various conditions (Figures 1 and S1). CADA induced a dose-dependent down-modulation of hCD4 regardless of the cell background in which it was expressed, i.e., in primary T-cells and T-cell lines that express the receptor naturally, as well as in transfected cells stably expressing CD4 (Figure 1B–1D). This down-modulating effect appeared to be reversible (Figure 1G), and selective for CD4: in a set of 14 different surface receptors CADA inhibited only CD4 (Figure 1E and 1F). The sensitivity of CD4 for CADA was species-specific: the compound did not affect the expression of mouse CD4, whereas primary T-cells from macaques responded to the compound in a similar way to human T-cells (Figure 1D). Thus, CADA selectively and reversibly down-modulates CD4 of primate origin in a dose-dependent manner.

### CADA Inhibits the Biogenesis of Human CD4 in a Signal Peptide-Dependent Way

**Figure 2:**
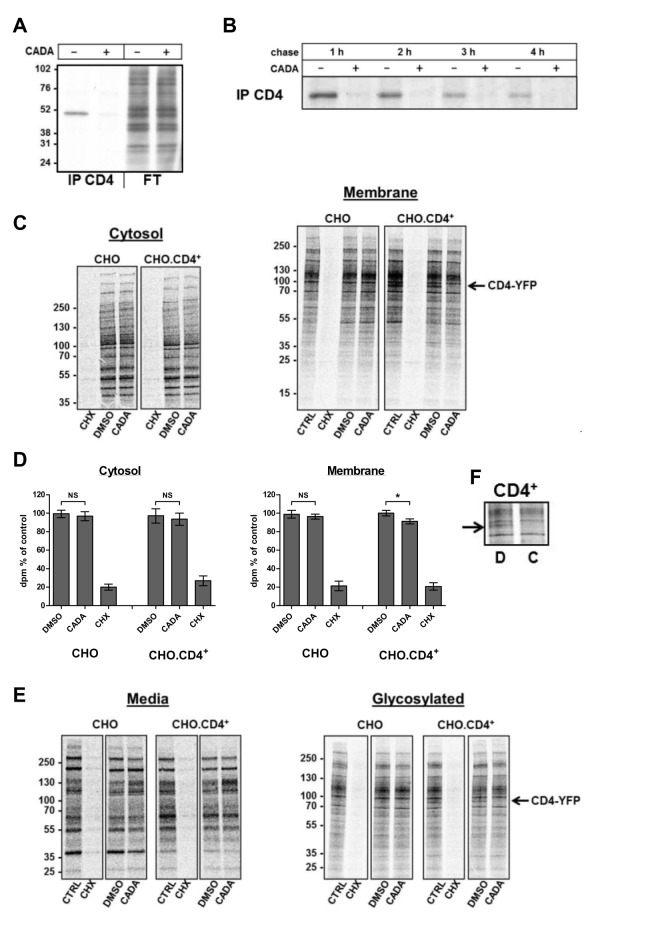
CADA specifically inhibits the biogenesis of human CD4. (A, B) CADA inhibits the biosynthesis of CD4. CD4^+^.CHO cells were washed and kept in methionine and cysteine-free medium in the presence or absence of 16 µM CADA for 45 min before exposure to [^35^S]methionine/cysteine (Met/Cys) for 30 min. Pulsed-labelled cells were then washed, lysed, and analyzed directly (A) or incubated in normal medium for up to 4 h (chase) in the presence or absence of 16 µM CADA (B). At specified time points cell lysates were immunoprecipitated for CD4. The flow through fraction (FT) of the CD4-immunoprecipitated samples is also presented. Note that the weaker CD4 bands in the control samples at longer chase time points are the result of the high turnover of hCD4 in CHO cells. Molecular mass is in kDa. (C–F) CD4 negative and stably CD4-YFP transfected CHO cells were pretreated with CADA (5 µM) or DMSO for 1 h before starvation in Met/Cys free medium with CADA, DMSO, or 50 µg/ml CHX. Cells were pulsed for 30 min, washed, and incubated in fresh medium without serum for 90 min. After collection of supernatant proteins (Media) cells were first permeabilized with digitonin buffer to obtain the cytosolic cell fraction before lysis in NP-40 buffer to collect the membrane proteins. Membrane fractions were further incubated with Concanavalin A (ConA) agarose beads (Glycosylated). Molecular mass is in kDa. (D) Quantification of ^35^S incorporation in (C) by scintillation counting (*n* = 4). NS, not significant; *p<0.01. (F) ConA fraction from a repeat experiment with CHO.CD4^+^ cells treated with DMSO or CADA. Note that the expression of CD4-YFP (∼80 kDa) is clearly reduced by CADA-treatment (indicated by arrow).

To elucidate how CADA interferes with CD4 protein expression, we analyzed the impact of CADA on the life cycle of CD4. A kinetic study with CADA and the phorbol ester PMA, a drug known to induce rapid endocytosis and degradation of surface CD4 [11], suggested an effect of CADA on CD4 translation or transport to the cell surface. As shown in Figure 1H, the appearance of newly synthesized CD4 molecules at the surface was prevented by CADA. To test directly whether CADA interferes with the *de novo* synthesis of CD4 we performed pulse-chase experiments followed by immunoprecipitation for CD4. CADA profoundly inhibited CD4 synthesis in CHO.CD4^+^ cells as indicated by the absence of this protein band in CADA-treated samples (Figure 2A and 2B). Although CADA strongly affected CD4, analysis of the total cellular protein extract revealed no general inhibition of protein synthesis (Figure 2A, lanes 3 and 4). We further analyzed this CD4-specificity of CADA by examining protein synthesis in different cellular compartments. Both cytosolic and membrane fractions were investigated in CD4-negative and CD4-positive CHO cells. The expression of cytosolic proteins was not altered by CADA-treatment, both in the absence or presence of CD4 (Figure 2C and 2D). In contrast, addition of the translation elongation inhibitor cycloheximide (CHX) resulted in an almost complete protein shut-down. Interestingly, CADA seemed not to affect other membrane proteins in the CD4-negative cells. Also, a similar membrane protein expression pattern was observed in DMSO and CADA-treated CD4^+^ CHO cells, except for one protein band migrating around 80 kDa. As this protein band could not be detected in the CD4-negative cells, we concluded that the affected protein was most likely CD4-YFP, an 80 kDa fusion protein that is stably and highly expressed in these CHO cells. The significant reduction in synthesis of membrane-associated proteins in CADA-treated CD4^+^ CHO cells (Figure 2D) can be ascribed to the complete block of CD4-YFP production. To isolate the glycosylated surface proteins from the membrane fraction that contains proteins not only from the surface membrane but also from different intracellular organelles and export pathways, we used Concanavalin A (ConA) agarose beads. Again, CD4-YFP was strongly inhibited by CADA, whereas the expression of other glycosylated membrane proteins was not affected (Figure 2E). In addition, CADA did not inhibit the secretion of proteins into the culture medium (Figure 2E). Similar observations were made in other CADA-treated cells, such as U87 and SupT1 (Figure S2). Finally, down-modulation of CD4 occurred post-transcriptionally, because CD4 messenger RNA levels were not altered by the compound (Figure S1F). From these data, we concluded that CADA has a high selectivity for the protein synthesis of hCD4.

**Figure 3:**
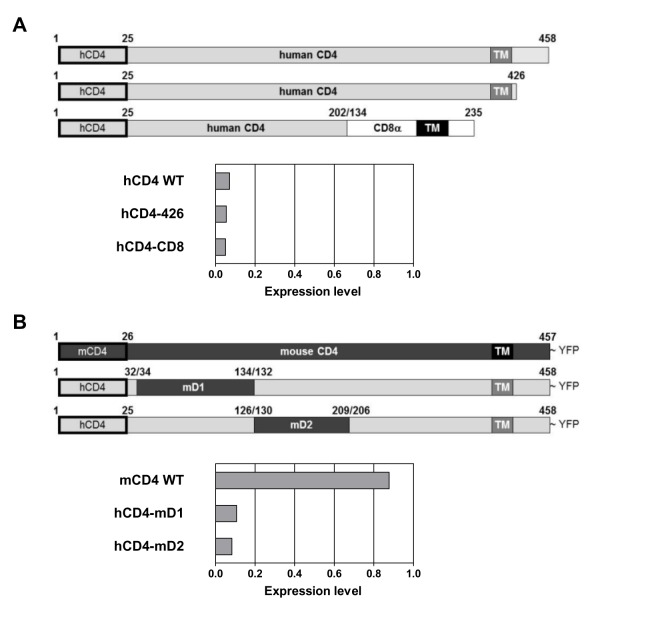
CADA inhibits the expression of human CD4 in a signal peptide-dependent way. (A) Schematic representation of the hCD4 C-terminal deletion constructs used. Numbers represent the corresponding amino acids of the pre-protein. The N-terminal SPs are indicated by a black rectangle. In mutant hCD4-426 the intracellular C-terminal domain has been removed. Construct hCD4-CD8 is composed of the D1 and D2 domains of hCD4 fused to the membrane-bound alpha chain of hCD8. CD4^−^ A2.01 human T-cells stably expressing hCD4WT, hCD4-426, or hCD4-CD8 were treated for 48 h with control medium or CADA (8 µM) and stained with anti-CD4 antibody to determine surface expression levels of WT or mutant CD4 by flow cytometry. Graph shows relative receptor expression levels of CADA-treated cells as compared to non-treated controls. (B) Schematic representation of the human/mouse chimaeric constructs used. Numbers represent the corresponding amino acids of the pre-protein. The N-terminal SPs are indicated by a black rectangle. The human/mouse chimaeric constructs are C-terminally fused to YFP for ease of flow cytometric detection. Construct hCD4-mD1 is composed of hCD4 in which the D1 region is replaced by the corresponding D1 domain of mouse CD4. It still contains the SP of hCD4 and the first seven residues of mature hCD4. Construct hCD4-mD2 is composed of hCD4 in which the D2 is replaced by the corresponding D2 domain of mouse CD4. HEK293T cells were transiently transfected with the expression plasmids and left either untreated or treated with CADA for 24 h. YFP fluorescence was determined and normalized to non-treated controls.

Next, we established which domain of CD4 is required for drug sensitivity by investigating C-terminal deletion mutants (Figure 3). Deletion of the cytosolic tail of CD4, which is involved in signal transduction and endocytosis of the receptor [11],[20], did not affect its sensitivity to CADA (Figure 3A, mutant hCD4-426). Exchanging the extracellular D3, D4, and transmembrane (TM) domains of CD4 with a related type I TM protein such as the alpha chain of CD8, expression of which is not affected by CADA (Figure 1F), failed to prevent CADA-induced down-modulation (Figure 3A, mutant hCD4-CD8). Furthermore, human/mouse chimaeric fusion constructs excluded a potential role for the immunoglobulin-like domains D1 and D2 of hCD4 in CADA-sensitivity. In agreement with primary murine T-cells (Figure 1D), wild-type mouse CD4 (mCD4 WT) did not respond to the down-modulating activity of CADA, whereas hCD4 containing either mouse D1 or mouse D2 did (Figure 3B). These data narrowed down the CADA-sensitive region of hCD4 to the N-terminal SP and the first seven N-terminal residues of the mature protein, as this is the only remaining fragment of hCD4 common to all CADA-susceptible constructs (Figures 3 and S1E).

### CADA Dose-Dependently Inhibits Translocation of Human CD4

**Figure 4:**
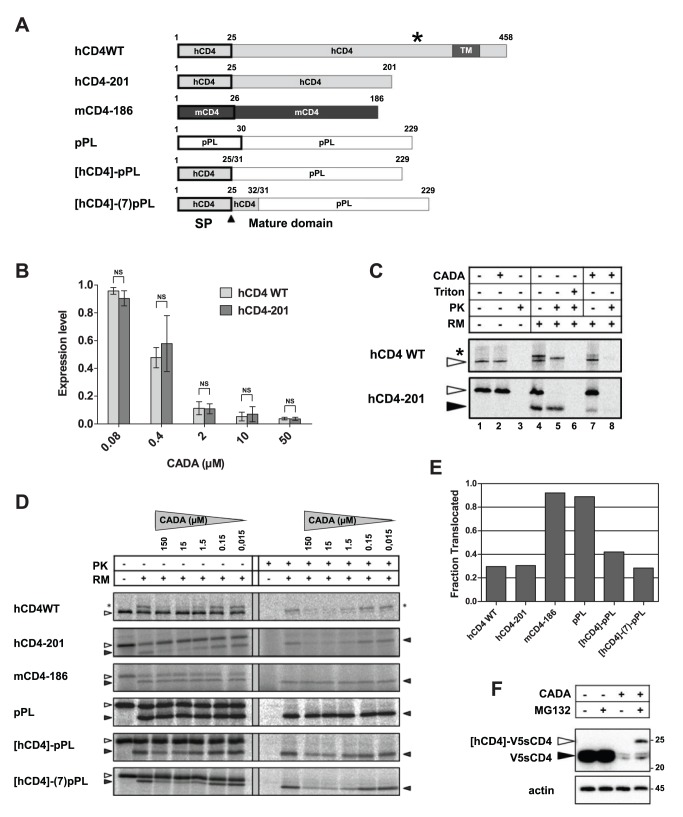
CADA dose-dependently inhibits co-translational translocation of human CD4. (A) Schematic representation of the constructs used. Numbers represent the corresponding amino acids of the pre-protein. The N-terminal SPs are indicated by a black rectangle. The star represents the N-glycosylation site in full length hCD4. (B) HEK293T cells were transiently transfected with the expression plasmids and left either untreated or treated with CADA for 48 h. Receptor expression levels were determined by flow cytometry and normalized to non-treated controls (*n*≥3). NS, not significant. (C) Transcripts encoding full length (hCD4 WT) and truncated (hCD4-201) hCD4 were translated in the presence of [^35^S]methionine and, where indicated, RMs and/or CADA (15 µM). Equal aliquots of the translated material were left untreated or treated with PK in the presence or absence of Triton X-100 (Triton). Samples were separated by SDS-PAGE and analyzed by autoradiography. The positions of precursor (open arrowhead), signal-cleaved (solid arrowhead), and glycosylated (asterisk) species are indicated. Of note is the lower appearance of the PK-protected glycosylated form of hCD4 WT (lane 5) compared to the non-PK form (lane 4), which is due to proteolytic cleavage of the 38 amino acid cytoplasmic domain, resulting in a smaller and faster migrating fragment. (D) As in (C), except that a 1∶10 serial dilution of CADA was used. (E) Translocation data of (D) quantified by phosphorimager analysis. The translocation efficiencies, at 15 µM CADA, normalized to control are indicated (mean values of at least three experiments). (F) Cytosolic degradation of precursor hCD4 in CADA-treated cells. HEK293T cells were transiently transfected with V5-tagged hCD4, treated with CADA (10 µM) and/or the proteasome inhibitor MG132 (200 nM). Cells were lysed in NP-40 buffer and analyzed by immunoblotting for CD4 with anti-V5 antibody. For the cell loading control, an anti-actin antibody was used. The predicted molecular mass for the SP-cleaved sCD4 (V5sCD4) was 22 kDa, whereas that of the precursor form ([hCD4]-V5sCD4) was 25 kDa. Molecular mass is in kDa. Similar data were obtained with 2 µM CADA. One representative experiment out of two is shown.

Previous experiments demonstrated an inhibitory effect of CADA on the expression of membrane-anchored proteins that contained the SP of hCD4. However, removal of the TM domain of CD4 did not diminish the sensitivity of the protein to CADA (Figure 4B), showing that CADA-susceptibility is not membrane anchor-dependent. Therefore, in our further study we also included smaller secreted proteins (Figure 4A).

In general, precursors of type I TM proteins (e.g., CD4) contain an amino-terminal SP that is involved in the early steps of biogenesis such as targeting of the nascent polypeptide to the ER membrane for co-translational translocation [21],[22]. To determine how CADA interferes with CD4 synthesis, translation and translocation of CD4 were analyzed *in vitro* using cell-free rabbit reticulocyte lysate with or without pancreatic rough microsomes (RMs). In the absence of RMs, translation of full length and truncated hCD4 was unaffected by CADA (Figure 4C, lanes 1 and 2, open arrowhead). However, in the presence of RM, translocation of hCD4 into the RM lumen was markedly inhibited by CADA as indicated by a reduction in glycosylated WT hCD4 (Figure 4C, lanes 4 and 7, star) and SP-cleaved truncated hCD4 (Figure 4C, lanes 4 and 7, solid arrowhead) that were resistant to degradation by added proteases (Figure 4C, lanes 5 and 8). In agreement with the *in cellulo* results of Figure 4B, the *in vitro* translocation of full length (hCD4 WT) and truncated secreted (hCD4-201) CD4 was dose-dependently inhibited by CADA (Figure 4D). In contrast, the translocation of two control molecules, mCD4-186 and bovine pre-prolactin (pPL), was not affected by CADA at any concentration tested (Figure 4D and 4E).

We next prepared two chimaeric constructs (Figure 4A) in which the mature domain of pPL was fused either directly to the SP of hCD4 ([hCD4]-pPL) or to the seventh residue of mature hCD4 ([hCD4]-(7)-pPL). Translocation of [hCD4]-pPL was profoundly inhibited by CADA (Figure 4D and 4E). While this mutant was slightly less sensitive to the compound than WT CD4, this confirms that sensitivity to CADA is determined primarily by the hCD4 SP. However, the presence of seven additional amino acids of the N-terminal CD4 mature domain enhanced CADA sensitivity, resulting in dose-dependent translocation inhibitory levels similar to WT CD4 (Figure 4E, construct [hCD4]-(7)-pPL). Furthermore, fusing these N-terminal 32 residues of hCD4 to a non-SP containing yellow fluorescent protein (YFP) resulted in translocation of a non-CD4 related cytosolic protein into the RM lumen and full susceptibility to CADA (Figure S3A–S3C). These data show that inhibition of protein translocation by CADA is specific to the SP and the first seven N-terminal residues of mature hCD4.

To investigate if CADA inhibits CD4 translocation into the ER *in vivo*, we performed experiments to rescue cytosolic CD4 in HEK293T cells by inhibiting proteosomal degradation with MG132. In order to detect all precursor forms of CD4, we generated an hCD4 construct that contained the simian virus 5 (V5) epitope at the N-terminal end of the mature protein (outlined in Figure S3E). Only when the cells were treated with the combination of CADA and the proteasome inhibitor MG132, a higher molecular form of CD4 could be detected that corresponded to the precursor form of the V5-tagged CD4 (Figure 4F). Accordingly, for [hCD4]-YFP proteasome inhibition with MG132 also rescued the precursor form in CADA-treated samples (Figure S3D). These *in vivo* results indicate that CADA diverts CD4 synthesis towards cytosolic proteosomal degradation.

### CADA Binds to hCD4 SP and Interacts Primarily with Its Hydrophobic H-Region

**Figure 5:**
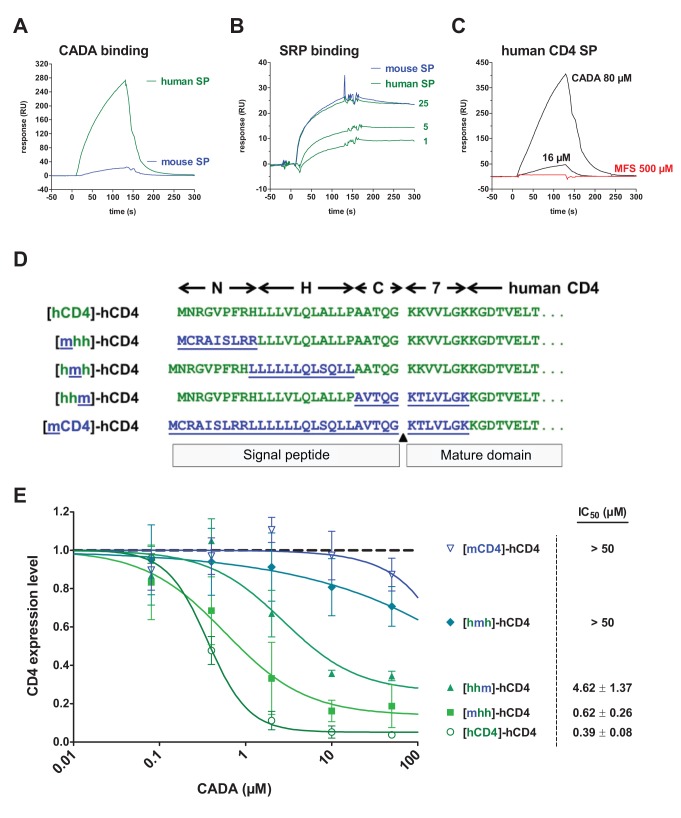
CADA binds to the signal peptide of human CD4 and interacts primarily with its hydrophobic H-region. (A) Binding of CADA to the SP of human but not to mouse CD4. Chemically synthesized peptides composed of the signal sequence and eight N-terminal residues of mature CD4, plus a PEG11 linker and a biotinylated lysine at the C-terminus, were captured on a streptavidin C1 chip for SPR analysis. The chip density was 84 and 153 resonance units (RUs) for human and mouse SP, respectively. Graph shows interaction of CADA (160 µM) to hCD4 SP (green) but not to mCD4 (blue). (B) As in (A), but for the binding of SRP to the SPs. The chip density was between 120 and 130 resonance units (RUs). Graph shows dose response of SRP (nM) to hCD4 SP (green). Similar binding of SRP (25 nM) was observed for mouse CD4 SP (blue). (C) The inactive CADA-analog MFS105 (MFS, red line) did not bind to the hCD4 SP (up to 1,000 µM), whereas for CADA a dose-dependent binding was measured (black lines). The chip density was 95 RUs. For clarity of the figure, only the 500 µM line is shown. Comparable data were obtained in an independent experiment shown in Figure S4D. (D) Schematic representation of the constructs used. The N-, H-, and C-regions of the SP of hCD4 were exchanged for those of mouse CD4. Also the first seven N-terminal residues of the mature protein were swapped as indicated. The residues of hCD4 are depicted in green, whereas those of mouse CD4 are in blue and underlined. (E) Flow cytometry analysis of HEK293T cells transiently transfected with the expression plasmids from (D) and left either untreated or treated with CADA for 48 h. Cells were collected and stained for hCD4. CD4 expression levels were normalized to non-treated controls (*n*≥3). The IC_50_ values of CADA for each construct are included.

We then looked for direct interaction between CADA and the hCD4 SP. Chemically synthesized SPs of hCD4 and mCD4 were captured on a streptavidin sensor chip and examined by surface plasmon resonance (SPR). Selective and dose-dependent binding of CADA to hCD4 SP was observed, and almost no binding to mCD4 SP (Figures 5A and S4A). Also, CADA did not show interaction with the SP of bovine pPL (Figure S4C). As a control, SRP interaction with the SPs was evaluated and revealed equal binding profiles of SRP to human and mouse SP, thus excluding non-functionality of mSP or insufficient peptide-coupling to the chip (Figures 5B and S4B). In addition, the lack of hCD4 SP binding by the structural analog MFS105 (Figure 5C), a CADA-derivative with no CD4 receptor down-modulating activity (Figure S4E) [8], further strengthens the selectivity of CADA for hCD4 SP.

SPs display a general structure consisting of three regions: a (mostly) positively charged N-terminus (N-region), a central hydrophobic alpha helical region (H-region), and a more polar C-terminal part (C-region) that includes the SP cleavage site [23]. On the basis of the unresponsiveness of mouse CD4 to CADA (Figures 1D, 3B, 4D, and 4E), we generated human/mouse chimaeric constructs with exchanged SP subregions in order to analyze the contribution of each subregion of hCD4 SP to CADA-susceptibility (Figure 5D). Exchanging the N-region of the SP (construct [mhh]-hCD4) slightly decreased the sensitivity of CD4 to CADA, as analyzed by flow cytometry (Figure 5E). A stronger reduction in susceptibility was observed when the C-region of mSP and the first seven N-terminal residues of mature mCD4 were inserted in hCD4 (Figure 5E, construct [hhm]-hCD4). Swapping the hydrophobic H-region had a major negative impact on the sensitivity to CADA (Figure 5E, construct [hmh]-hCD4). Reversibly, insertion of the hydrophobic alpha helix of hCD4 SP into CADA-insensitive SPs, such as those of murine CD4 or bovine pPL, significantly enhanced their responsiveness to CADA (Figure S5C and S5D). In addition, the different inhibitory levels of CADA on surface expression of the human-murine chimaeras could be linked to different degrees in ER translocation inhibition (Figure S5A and S5B). Although a more than 10-fold reduction in CADA-activity was noted in the *in vitro* assay as compared to the *in cellulo* data (Figure 5E versus S5B), the chimaeras responded to CADA in the same relative order. These data suggest contributions from all three hCD4 SP subregions to CADA-sensitivity, but show a crucial role for the hydrophobic H-region.

### CADA Inhibits the Translocation of [CD4]-pPL Nascent Peptide Chains at a Post-Targeting Step

**Figure 6:**
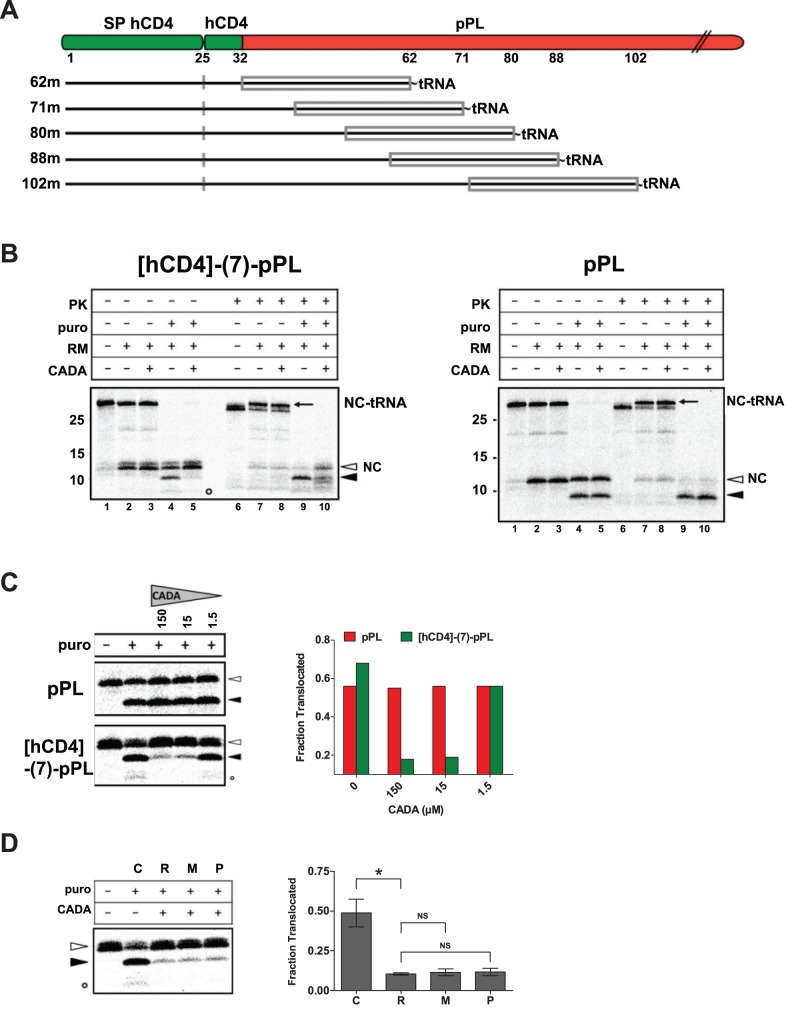
CADA inhibits co-translational translocation of hCD4-pPL at a post-targeting step. (A) Schematic representation of the truncated nascent chains of [hCD4]-(7)-pPL. The nascent chains remain attached to the ribosome through a peptidyl-tRNA bond. The (approximately) 30 residues at the C-terminus of the polypeptide that are buried inside the ribosome are indicated by a grey rectangle. The SP cleavage site (at residue 25) is also marked. (B) *In vitro* translation, targeting, translocation (puromycin-release), and PK digestion of [^35^S]methionine-labelled [hCD4]-(7)-pPL 80-mers (left panel) and WT pPL 78-mers (right panel). Intact peptidyl-tRNA bands (NC-tRNA, arrow) are indicated and represent the targeted and PK protected RNCs. Note that the presence of microsomes (RM) releases some nascent chains (NC) from tRNA in an unproductive way (compare lanes 1 and 2, open arrowhead). The positions of released precursor (open arrowhead) and signal-cleaved (solid arrowhead) polypeptide chains are indicated. The SP of hCD4 but not pPL was also detected (open circle). Molecular mass is in kDa. (C) Dose response of CADA (µM) on translocation of RNCs as in (B). Graph shows translocation fractions of the autoradiogram quantified by phosphorimager analysis. (D) Time of addition of CADA. [hCD4]-(7)-pPL nascent chains of 80 residues (80-mers) were synthesized in the absence of membranes before exposure to RM for targeting. Nascent chains were left untreated (C, control), or were treated with CADA (15 µM) either administered at the beginning of the synthesis in the translation mixture (R, ribosomes), administered to membranes for pretreatment (M, microsomes), or applied to the RNC/RM mixture 15 minutes after initiation of targeting but before puromycin release (P, post-targeting). Graph shows translocation data of three experiments quantified by phosphorimager analysis. NS, not significant. *p<0.01.

To identify the step at which CADA interferes with the translocation of hCD4, we analyzed the targeting and translocation of [CD4]-pPL nascent chains. Transcripts lacking a stop codon were truncated at sequential sites in the mature domain (Figure 6A) and translated *in vitro*. Via an intact peptidyl-tRNA bond the nascent chains remain attached to the ribosomes and are synchronized at a defined length. Addition of pancreatic RMs will result in docking of the different ribosome-nascent chain complexes (RNCs) to the ER membranes, giving translocation intermediates that represent static snapshots of the movement of the SP within the translocon channel. Subsequently, the nascent chains are released from membrane-bound ribosomes by puromycin, allowing for a controlled translocation into the ER lumen.

Nascent chains of 80 residues (80-mers) were translated in the absence of RM (Figure 6B, left panel, first lane). These chains are bound to the ribosome as peptidyl-tRNAs with about 30 residues buried inside the ribosome. Protease treatment of these RNCs will degrade the N-terminal part of the peptide that is exposed to the cytosolic compartment (about 50 residues), and will generate a faster migrating protein band on the gel (Figure 6B, lane 6). Addition of RM to the nascent chains will allow for targeting of the RNCs to the membrane and insertion of the SP into the translocon channel. If well-targeted, nascent chains will be shielded from exogenous protease because of a tight interaction between the ribosome and the translocon after transfer from SRP [24],[25], and appear as intact RNCs after proteinase K (PK) treatment. Equal protease-protected peptidyl-tRNA bands were observed in control and CADA samples, ruling out an inhibitory activity of CADA on targeting and transfer of the nascent chains to the translocon (Figures 6B, lanes 7 and 8, arrow). Addition of puromycin to the targeted chains induced the release of the nascent chains from the ribosome, with subsequent translocation of the peptides into the PK-protected RM lumen and cleavage of the SP (Figure 6B, lanes 4 and 9, solid arrowhead). However, in the presence of CADA, very few cleaved species were observed (Figure 6B, left panel, lane 5, solid arrowhead), indicating a profound inhibition by CADA on the co-translational translocation of pPL species that contained the SP of hCD4 but not of those with the SP of WT pPL (Figure 6B, left and right panel, respectively). This inhibition was also dose-dependent (Figure 6C). Translocation inhibition by CADA was observed at all chain lengths, but was most effective on nascent chains up to 80 residues (Figure S7).

Remarkably, we could delay the administration of CADA to the membranes until targeting was completed. A similar inhibitory effect on the translocation of 80-mers was recorded when the compound was applied to the RM before targeting or to the RNC/RM mixture 15 minutes after initiation of targeting (Figure 6D). Taken together, these results indicate that CADA acts at a step after the targeting and transfer of the nascent chains to the translocon, but before the growing peptide chain has reached the luminal side of the membrane.

### CADA Interferes with the Topology Inversion of hCD4 SP

**Figure 7:**
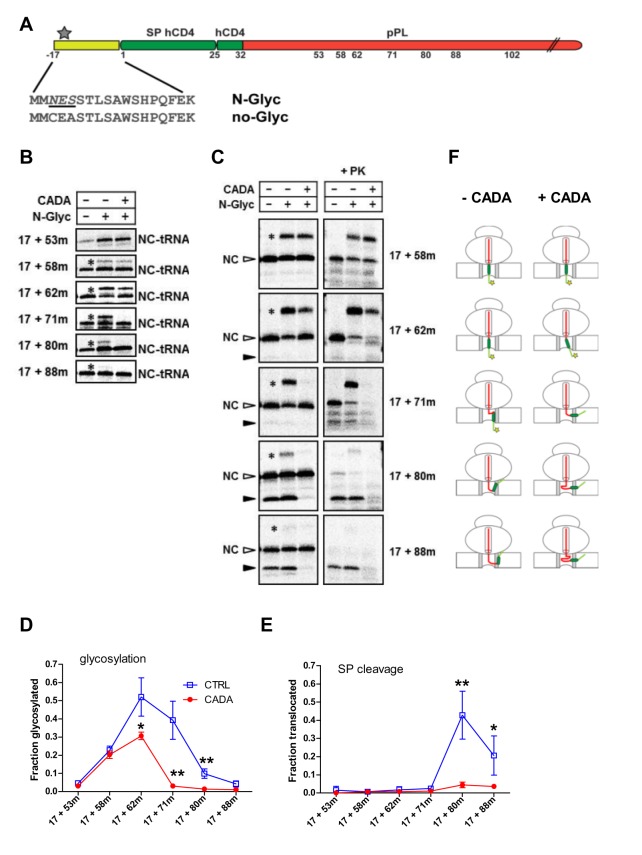
CADA locks the hCD4 SP in an intermediate inversion conformation in the translocon. (A) Line diagram of the constructs with an N-terminal extension of the SP lacking (no-Glyc) or containing a diagnostic glycosylation site (N-Glyc). For ease of comparison, the numbering refers to the non-extended WT hCD4 SP-containing polypeptide. The N-glycosylation site is underlined and the star indicates the N-glycosylated form of the protein. (B) Autoradiogram of truncated *in vitro* translated [^35^S]methionine-labelled [hCD4]-(7)-pPL RNCs with the glycosylation site-containing extension (N-Glyc). A non-glycosylation control (no-Glyc) is included (every first lane). Nascent chains were synthesized in the presence of RM, with or without CADA (15 µM). Samples were separated by SDS-PAGE and analyzed by autoradiography. Intact RNCs (NC-tRNA) and N-terminal glycosylated RNCs (asterisk) are indicated. Notice that CADA strongly inhibited the glycosylation of the 17+71-mer. (C) Same as in (B), but for the puromycin-treated samples. Equal aliquots of the translated material were left untreated or treated with PK. Released non-processed nascent chains (NC, open arrowhead), N-terminal glycosylated forms (asterisk) and signal-cleaved C-terminal translocated species (solid arrowhead) are indicated. (D) Fraction of glycosylated nascent chains in the absence or presence of CADA as in (C), quantified and plotted against chain length (*n*≥3). *p<0.05; **p<0.001. (E) Fraction of SP-cleaved C-terminal translocated nascent chains in the absence or presence of CADA as in (C), quantified and plotted against chain length (*n*≥3). *p<0.01; **p<0.001. (F) Schematic of ribosome/translocon complexes showing probable positioning of the SP of hCD4 in absence or presence of CADA. Star represents N-glycosylation.

Although N-terminal signal sequences are generally considered to insert in a tail-first, hairpin-looped topology [26],[27], hydrophobic sequences can also enter the translocon in a head-first configuration or reorient within the translocon channel [28]–[32]. To determine if CADA might have a role in changing the topology of the SP during translocation, we introduced a diagnostic N-glycosylation site at the N-terminus of the SP (Figure 7A). Head-first N-terminal translocation of the SP will give rise to glycosylated species, whereas tail-first C-terminal orientation will result in SP-cleaved forms.

The hCD4 SP was extended with 17 residues based on a construct used in other studies [33],[34], and either contained or lacked (control) an N-linked-glycosylation site (Figure 7A). Full length proteins with the extended hCD4 SP translocated well, irrespective of the presence of the glycosylation site, and responded to CADA in a dose-dependent way (Figure S8A). Through analysis of nascent chains we investigated if and when head-first translocation occurred, and determined the stepwise movement of the extended SP within the translocon (Figure 7B). At shorter truncations (i.e., 17+58-m), a substantial fraction of the nascent chains were glycosylated, indicating that targeting may begin with the N-terminus of the SP in the ribosome/translocon complex facing the RM lumen. At this chain length, the binding of the RNC to the translocon was already highly stable as evidenced by its high-salt resistance (Figure S8E). Elongation of the mature part of the polypeptide with four residues (17+62-m) resulted in a profound increase in glycosylated RNCs (Figure 7B, second lane of third panel, star). At longer truncations, the glycosylation efficiency decreased gradually and was almost undetectable for the 17+88-mer. Interestingly, administration of CADA to the RNCs had very little effect on the N-terminal glycosylation of the shorter chains (17+58-m) and the high-salt resistant binding of the RNC to the translocon (Figure 7B, third lane of second panel, and Figure S8E). However, the inhibitory activity of CADA increased significantly when the C-terminus of the polypeptide was extended, and resulted in non-detectable glycosylation levels for chains with a minimum length of (17+71) residues (Figure 7B). Notably, administration of CADA to the 17+71-mers resulted in a loss of the high-salt resistant binding of the RNC to the translocon (Figure S8E), suggesting that with the compound the positioning of the nascent chain in the channel was altered so that the peptide tether could allow the ribosome to dissociate from the channel. Analysis of the puromycin-treated samples revealed a similar N-terminal glycosylation pattern for the released chains as for the intact RNCs (Figure 7C and 7D). Again, the inhibitory activity of CADA on N-terminal glycosylation gradually increased with growing chain length for chains up to (17+71) residues (Figure 7D). Breaking the peptidyl-tRNA bond with puromycin also allowed for translocation of the chains into the RM lumen with subsequent SP cleavage. SP-cleaved species were hardly detectable for the shorter chains, irrespective of the presence of a glycosylation site (Figure 7C and 7E, blue line). However, translocated SP-cleaved chains that were PK-protected first appeared for the 17+80-mers, i.e., a chain length at which a profound decrease in N-terminal glycosylation was noted (Figure 7C–7E). This suggests that at this specific chain length the SP is mainly positioned in the hairpin-looped topology with the C-terminus facing the RM lumen. In accordance with the data from the WT SP-containing chains (Figure S7B), maximum C-terminal translocation and SP-cleavage were noted for the 17+80-mers, thus when the distance from the SP cleavage site to the ribosome peptidyltransferase centre (PTC) had reached a chain length of about 55 amino acids. Further extension of the polypeptide chain diminished the C-terminal translocation efficiency that was consistently blocked by CADA (Figure 7E). Notably, for the 17+80-mers CADA strongly inhibited both the (weak) N-terminal glycosylation and the C-terminal translocation and SP-cleavage with equal efficiency, in a dose-dependent manner (Figure S8C and S8D).

These results together show that the nascent chains need a minimum length in order for CADA to exert its inhibitory effect on glycosylation, whereas at all lengths where SP-cleavage can be observed translocation with SP-cleavage is inhibited. Thus, on one hand, CADA hinders vertical positioning of the polypeptide with the N-terminus faced to the ER lumen for efficient glycosylation and, on the other hand, disturbs the completion of SP inversion for a hairpin-looped structure that can be SP-cleaved (Figure 7F). This suggests that in the presence of CADA the SP is held in a folded conformation in the translocon channel, so that the polypeptide is prevented from reaching the luminal side of the ER.

## Discussion

In this study we have characterized CADA, a small-molecule HIV entry inhibitor as, to our knowledge, the first SP-binding drug that selectively inhibits hCD4 protein translocation into the ER in a SP-dependent way.

CD4 is a type I membrane protein expressed on the surface of a subset of immune cells [1]. Sorting of this protein to the plasma membrane requires that the CD4 pre-protein contains a cleavable SP, an obligatory component for protein targeting to the ER through co-translational translocation. This synthetic pathway begins when a hydrophobic N-terminal SP emerges from the ribosome, is recognized by the SRP, and targets the whole RNC to the ER membrane so that the emerging polypeptide is inserted into the Sec61 translocon channel [16],[17]. Simultaneously with the translocation of the polypeptide chain, cleavage of the SP occurs at the luminal side of the ER. Here, we revealed that targeting of hCD4 to the ER is initiated with a head-first N-terminal insertion (N_lum_/C_cyt_) of the SP in the translocon, before it inverts into an obligate N_cyt_/C_lum_ topology necessary for SP cleavage during C-terminal translocation. Thus, initial insertion of hCD4 SP in the channel occurs differently from the more widely accepted hairpin positioning (N_cyt_/C_lum_) of cleavable N-terminal signals [26],[27],[35]. Head-first topology of non-cleavable N-terminal sequences has been proposed by the Spiess group for signal-anchor (SA) sequences that anchor the polypeptide in the bilayer [29]–[31],[36]. Recently, a detailed study with an N-terminal type II SA sequence confirmed the head-first insertion of the SA into Sec61α before inversion from type I to type II topology takes place [32]. It is plausible that cleavable N-terminal SPs insert and invert in a similar way as type II SA sequences. Moreover, for a relatively short nascent polypeptide that is C-terminally attached to the ribosome PTC, the first possible way for the N-terminal SP to interact with the translocon is most likely a head-first (N_lum_/C_cyt_) orientation. Initial positioning of signal sequences may also be directed by interaction with cytosolic chaperones other than SRP, and translocon-associated proteins. Recently, Sec62 has been found to mediate the orientation of SA proteins depending on the hydrophobicity of the SA sequence [37]. It would be interesting to explore if there are other type I membrane proteins with initial N_lum_/C_cyt_ insertion and to investigate if the factors that determine this early positioning of cleavable SP are different from those reported for N-terminal SA sequences.

Reorientation of SA sequences is driven by flanking charges according to the positive-inside rule [38],[39] and inhibited by increased hydrophobicity of the SA sequence [40]. For hCD4 the two adjacent lysine residues immediately down-stream of the SP cleavage site might overrule the more dispersed positive charge of the N-domain (two arginine residues R3 and R8) and initially orient the SP according to this positive-inside rule with its C-terminus to the (negatively charged) cytosolic side of the membrane. In line with the canonical SA derived from the first TM segment of aquaporin 4 [32], hCD4 SP initiates ER targeting at a nascent chain length of 58 residues, when the size of the polypeptide between the SP C-terminus and the PTC is about 33 residues. Inversion of hCD4 SP and translocation of its C-terminal segment with SP-cleavage were observed upon lengthening the polypeptide chain (at its C-terminus) with 22 amino acids, a stretch theoretically long enough to span the bilayer membrane and thus to allow for a hairpin looped positioning of the SP. For our study we extended the N-terminal hydrophilic domain of hCD4 SP with 17 residues as this was the minimum length required to obtain optimal glycosylation of the N-terminus (Figure S9A–S9C), because of the distance between the active site of the oligosaccharyl transferase complex and the inner bilayer of the ER membrane [41]. Extension of the N-domain of SA sequences with more than 20 residues can change their signal insertion behaviour, but in favour of C-terminal insertion [36]. Our extended hCD4 SP did not shift to a preferentially C-terminal translocation with SP-cleavage (Figure S9D), suggesting that the extended hCD4 SP preserved the same insertion behaviour as WT SP.

Our data indicated equal targeting efficiencies for control and CADA-treated nascent chains, making interference of CADA with SRP-binding less likely. Furthermore, the equal mRNA levels that were detected in CADA-treated and control cells indicate that the compound does not induce an mRNA degradation response as has been observed with defective signal sequence recognition by SRP [42]. Also, early insertion of the SP into the translocon channel was not altered by our drug, ruling out inhibition of channel gating by CADA. The post-targeting translocation inhibition by CADA is probably related to the obligate inversion of hCD4 SP inside the translocon. One can expect that the reorientation of the hCD4 SP from an N_lum_/C_cyt_ into an N_cyt_/C_lum_ topology requires a high degree of flexibility of the SP and the translocation channel to undertake such a gymnastics, which may be compromised after CADA-binding. Based on the CADA-sensitive chimaeras that contained the hydrophobic alpha-helix of hCD4 SP, we could attribute a crucial role to the core hydrophobic domain of the SP and hypothesize compound binding to this region. In fact, minor changes in this alpha-helix, such as removing the helix terminator Pro20, could already reduce CADA-dependency, but introducing this proline residue at the C-terminal part of the murine ortholog was not sufficient to render sensitivity to the drug (see Figure S6). However, not only the central hydrophobic α-helical H-region of hCD4 SP, but also its C-terminus together with the first residues of the mature protein was shown to be important for full CADA-sensitivity. Interaction of CADA with both regions might re-position the SP in the translocon and profoundly reduce flexibility or alter the balance between hydrophobic and electrostatic interactions of the SP with the translocon and the lipid environment [43]–[45]. Also, post-targeted folding of a zinc-finger modified pPL has recently been shown to inhibit co-translational translocation into ER microsomes, indicating that folding events within the ribosome-Sec61 translocon complex (RTC) can occur and induce a mechanical block within the RTC that diverts the nascent chain into the cytosol for degradation [46]. Accordingly, in the presence of CADA the non-translocated CD4 precursor forms are routed to the cytosol for proteasomal degradation, which also indicates that translocation and not translation of CD4 is inhibited by the compound.

In our experiments, C-terminal translocation of nascent chains was determined by SP-cleavage. Inhibition of the signal peptidase activity could also be interpreted as an inhibition of translocation, however, CADA did not interfere with the enzymatic activity of RM-extracted signal peptidase (Figure S9E). Furthermore, both inhibition of N-glycosylation and SP-cleavage were observed with the N-terminally extended 80-mers, making it very unlikely that CADA would inhibit both luminal enzymes that act at distinct parts of the SP. Therefore, we propose that in the presence of CADA the SP is held in a folded conformation in the translocon (presumably at the lateral gate), so that the polypeptide is prevented from moving through the channel.

The focus of this work was to unravel the mechanism through which CADA selectively regulates CD4 expression. We show that CADA selectively inhibits the biogenesis of CD4 in cells without affecting other membrane or secretory proteins, and that this is due to its ability to inhibit specifically the co-translational translocation of CD4 into the lumen of the ER. The activity of CADA maps to the cleavable N-terminal SP of hCD4. Moreover, through SPR analysis we were able to show direct binding of CADA to the SP of hCD4 and identify this SP as the main target of our drug. In view of the very high selectivity of CADA for hCD4, it is most plausible to hypothesize a unique SP as its target. However, we cannot completely exclude a secondary interaction of CADA with another target of the translocation machinery. This could be in line with the suggested bimolecular binding model for CADA based on data from unsymmetrical CADA analogs and the 3-D quantitative structure-activity relationship (3D-QSAR) study [47],[48]. If CADA interacts with Sec61 (or closely related factor) in a more unspecific and non-discriminating manner, the final decision for a substrate to become translocation defective would then depend on the degree of reduced SP-flexibility by CADA binding, suggesting that CADA-dependency would mostly rely on SP-recognition and binding.

Hydrophobic signal sequences, such as SPs, perform several functions in the biogenesis of secretory and membrane-bound proteins [26]. Although these hydrophobic sequences may represent interesting targets for drug design in order to regulate the expression level of proteins, CADA is the first small-molecule drug, to our knowledge, known to selectively bind to a SP and inhibit translocation of a specific protein in a SP-dependent way. There is precedence for the cyclic heptadepsipeptide HUN-7293 and its derivatives CAM741 and cotransin to down-modulate vascular cell adhesion molecule 1 (VCAM1) in a signal-sequence-discriminatory way [49],[50]. However, these compounds were reported to bind to the Sec61 translocon channel and to inhibit the expression of a subset of secretory and membrane proteins, including VCAM1 [34],[51]–[53]. In contrast, the expression levels of a range of cell surface receptors including VCAM1 (Figure 1F) and CD4 from a different (non-primate) species (Figure 1D), were not decreased by CADA, which is explained by the interaction of the drug with a SP of a specific cell surface receptor instead of a common Sec61 channel. Recently, the cyclic dodecadepsipeptide valinomycin has been described to selectively destabilize the SP of hamster prion protein [54]; however, this might be related to the down-regulation of the luminal chaperone protein BiP involved in protein translocation, explaining the general apoptosis-inducing effect of this drug [55]. The toxic, small-molecule eeyarestatin 1 (ES_I_) has been reported to inhibit co-translational translocation of a wide-range of receptors, but this compound prevents the transfer of the nascent polypeptide chain from the membrane-bound SRP delivery complex to the ER translocon, thus acting more upstream of CADA and the cyclodepsipeptides and less substrate-selective [56]. Mycolactone, the virulence factor of *Mycobacterium ulcerans* has recently been described to block protein translocation into the ER, and thus, to prevent secretion of innate cytokines and expression of important immune-related membrane receptors [57]. Also, apratoxin A, a natural product from a marine cyanobacterium prevents co-translational translocation of a wide-spectrum of cellular proteins, which may also explain its cytotoxic nature [58]. Cellular cytotoxicity is a major concern when using small synthetic drugs; however, cellular viability was not compromised by CADA and long-term (∼1 year) CADA-treatment of T-cells was achieved, with preservation of CD4 re-expression following workout.

In conclusion, our findings demonstrate that with cell-permeable small synthetic compounds selective SP-binding and subsequent translocation inhibition of the associated protein is feasible. This opens a new field, not only for CD4 related immunomodulation and antiviral intervention, but also for a wide range of cell surface proteins for which this principle of selective SP-targeted translocation inhibition should be applicable.

## Materials and Methods

### Compound

CADA.HCl and MFS105.HCl were synthesized as described previously [47]. The compounds were dissolved in DMSO and stored at room temperature in the dark.

### Constructs

The pcDNA3 expression vector (Invitrogen) encoding WT hCD4 was kindly provided by O. Schwartz (Institut Pasteur, Paris, France). The A2.01/hCD4.426 and A2.01/hCD4-CD8 cell clones were from C. Devaux (Montpellier, France) and have been described elsewhere [59]. The pReceiver-M16 vector encoding mouse CD4-YFP was purchased from Imagenes and the human-mouse chimera constructs [60] were kindly provided by A. Trkola (Zurich, Switzerland). Bovine pPL in the pGEM4 vector has been described previously (plasmid pGEMBP1 [61]). Human and mouse CD4 were cloned into the pGEM4 vector for *in vitro* translation experiments. Fusion constructs were generated by PCR and cloned into the pGEM4 vector. The human/mouse chimaeric constructs were generated by incorporating the mouse sequence into the coding human sequence using PCR overlap extension and site-directed mutagenesis (Stratagene). The same strategy was used for the murine/hCD4 and pPL/CD4 chimaeras. For the V5-tagged CD4 construct [hCD4]-V5sCD4, the simian virus 5 (V5) epitope (GKPIPNPLLGLD) was inserted in the pcDNA3.1-hCD4D1D2 plasmid, which contains the coding sequence of the two N-terminal domains of the hCD4 protein. The V5 sequence was incorporated at the N-terminus of mature CD4 between residue 32 and 33 as schematically presented in Figure S3E. Note that the first two residues of V5 (residue G and K) were already present in CD4 at positions 31 and 32.

### Cell Culture and Cellular Assays

The CD4^+^ T-cell lines MT-4 and SupT1 were cultured in RPMI-1640 medium, and Hela, U87, HEK293T, and CHO cells were cultured in DMEM, supplemented with 10% FCS and penicillin/streptomycin. Human PBMCs were isolated by density gradient centrifugation as described previously [18]. For the monkey PBMCs, blood was collected from cynomolgus macaques (*Macaca fascicularis*) as described elsewhere [62]. Murine PBMCs were isolated from blood collected from Balbc mice. Stable transfections of U87 cells were performed with FuGENE 6 Transfection Reagent (Roche Diagnostics), and transient transfections of HEK293T cells were performed with PolyJet *in vitro* transfection reagent (Tebu-Bio), in accordance with the manufacturer's instructions. Western blot analysis of cell lysates was performed in accordance with standard protocols. Mouse monoclonal antibodies anti-hCD4 (clone SK3, BD Biosciences), anti-clathrin heavy chain (clone 23, BD Biosciences), anti-THE SV5-tag mAb (Genscript), anti-actin (Abcam ab3280), and a HRP-conjugated goat anti-mouse antibody (Dako) were used for detection.

For the cytosolic CD4 precursor rescue experiments, HEK293T cells were transfected with a mixture of 500 ng pcDNA3.1-V5-hCD4D1D2 plasmid DNA and 2 µl lipofectamine 2,000 (Invitrogen). Six hours after transfection, growth medium was removed and replaced with fresh medium containing 10 or 2 µM CADA and/or 200 nM MG132 (Sigma). After a 22 hour incubation in the presence of the compounds, cells were washed with ice-cold PBS and lysed in ice-cold lysis buffer (25 mM Tris, 150 mM NaCl, 1 mM EDTA, 1% NP-40, 5% glycerol, pH 7.4), supplemented with 0.4 µM PMSF (Fluka) and Complete protease inhibitor cocktail (Roche).

### Flow Cytometry

Flow cytometric analysis of surface receptor expression was performed as described previously [18]. All FITC-, PE-, PerCp-, or APC-labelled mAbs were from BD Biosciences. Data were acquired with a FACSCalibur flow cytometer (BD Biosciences) and the CellQuest software (BD Biosciences). Data were analyzed with the FLOWJO software (Tree Star). Down-modulation of CD4 was evaluated by the decrease in fluorescence intensity on CADA-treated cells relative to matched, untreated cells stained for CD4. To calculate the efficiency of CADA on CD4 down-modulation, the median fluorescence intensity (MFI) for CD4 labelling for each sample was expressed as a percentage of the MFI of control cells (after subtracting the MFI of the unstained control cells).

### Metabolic Labelling

Pulse-labelling of CD4 was performed on CD4^+^.CHO cells preincubated for 45 min at 37°C with CADA (16 µM) in medium lacking methionine, cysteine, and serum. After being pulsed with 0.75 mCi ml^−1^ [^35^S]methionine/cysteine for 30 min, cells were chased by replacing the radiolabel with pre-warmed medium containing 1 mM cysteine and 1 mM methionine. At the end of the chase this medium was replaced by ice-cold DMEM. Cells were kept on ice and lysed in NP-40 buffer (2% NP-40, 20 mM Tris [pH 7.8], 150 mM NaCl, 2 mM MgCl_2_), and cell lysates (separated from nuclei and debris) were immunoprecipitated for CD4 as described [63]. Proteins were analyzed by SDS-PAGE and autoradiography.

### Protein Synthesis in Different Cellular Compartments

Cells were pretreated with CADA (5 µM) or DMSO for 1 h before starvation in Met/Cys free medium with CADA, DMSO, or 50 µg/ml CHX (Sigma). Cells were pulsed with 0.022 mCi ml^−1^ [^35^S]methionine/cysteine EasyTag Protein Labeling mix (Perkin Elmer) for 30 min, washed twice with PBS, and incubated in fresh medium without serum for 90 min. After collection of the supernatant, cells were washed in ice-cold PBS and then either lysed in ice-cold lysis buffer (25 mM Tris, 150 mM NaCl, 1 mM EDTA, 1% NP-40, 5% glycerol, pH 7.4) supplemented with 0.4 µM PMSF and protease inhibitor cocktail, or separated into cytosolic and membrane fractions. To collect the cytosolic proteins, cells were permeabilized with digitonin buffer (20 mM Tris, 150 mM NaCl, 2 mM MgCl_2_, 0.03% digitonin, pH 7.8, supplemented with 0.4 µM PMSF and protease inhibitor cocktail). Permeabilized cells were then washed in digitonin buffer and further lysed in ice-cold lysis buffer (25 mM Tris, 150 mM NaCl, 1 mM EDTA, 1% NP-40, 5% glycerol, pH 7.4, supplemented with 0.4 µM PMSF and protease inhibitor cocktail). To isolate glycosylated proteins, total cell lysates or digitonin-resistant membrane fractions were further incubated with Concanavalin A (Vector Laboratories) agarose beads overnight at 4°C by gentle rotation.

### Cell-Free Translation

Full length cDNAs and truncated CD4-pPL nascent chains generated by PCR were transcribed *in vitro* using T7 RNA polymerase, and translated in rabbit reticulocyte lysate (Promega) in the presence of [^35^S]methionine (Perkin Elmer). Translations were performed at 25°C for 45 min (full length) or for 25 min (truncated) in the presence or absence of mammalian pancreatic microsomes (RMs). Digestion with Proteinase K (Roche) was performed on ice for 30 min and stopped by the addition of phenylmethylsulfonyl fluoride (PMSF; Thermo Fisher Scientific). Release of the nascent chains (NCs) from the targeted ribosome was induced by treatment with 2 mM puromycin for 10 min at 25°C. NCs were isolated by sedimentation at 4°C and pellets were dissolved in SDS sample buffer for analysis by SDS-PAGE. For the quantitative analysis of translocation experiments we used a Cyclone Plus phosphorimager (Perkin Elmer) with accompanying software.

For the time-of-addition experiments, [hCD4]-(7)-pPL nascent chains of 80 residues were synthesized in rabbit reticulocyte lysate by translating the mRNA for 20 min at 25°C. Translation was done in the absence of RM, and the translation mixture was left untreated (C, control) or was treated with CADA (15 µM) from the beginning of the translation reaction (R, ribosomes). In the mean-time, some RMs were pretreated with CADA (15 µM). After 20 min of translation, the untreated control sample was split in three aliquots. Next, one aliquot of control sample and the CADA-sample were given untreated RMs. The CADA-pretreated RMs were administered to the second control sample (M, microsomes). The third control sample received untreated RMs. All samples were then incubated for 10 min on ice and 5 min at 25°C. Finally, CADA was administered to the third control sample (P, post-targeting), and all samples were further incubated for 5 min at 25°C, before treatment with puromycin. NCs were isolated by sedimentation at 4°C and pellets were dissolved in SDS sample buffer for analysis by SDS-PAGE.

### Surface Plasmon Resonance Analysis

Chemically synthesized SPs (PEPperPRINT) were captured on a streptavidin-coupled C1 sensor chip (GE Healthcare). The amino acid sequence of the SP was: MNRGVPFRHLLLVLQLALLPAATQGKKVVLGKK-PEG11-K-biotin for hCD4, MCRAISLRRLLLLLLQLSQLLAVTQGKTLVLGKE-PEG11-K-biotin for mCD4, and MDSKGSSQKGSRLLLLLVVSNLLLCQGVVSTPVCPNGP-PEG11-K-biotin for pPL. The chip density was between 84 and 215 resonance units (RUs). A reference flow cell was used as a control for non-specific binding and refractive index changes. All interaction studies were performed at 25°C on a Biacore T200 instrument (GE Healthcare). The compound CADA was diluted in HBS-P (10 mM HEPES, 150 mM NaCl, and 0.05% surfactant P20 [pH 7.4]) supplemented with 5% DMSO. Signal recognition particle (tRNA Probes) was included as a positive control. Samples were injected for 2 min at a flow rate of 30 µl/min and the dissociation was followed for 2 min.

### Statistics

All data are presented as means with standard deviations (SD), unless otherwise stated. Two-tailed Student's *t* test was used to determine statistical significance as calculated with GraphPad software. For the flow cytometric data, statistical analysis was done on the background-corrected MFI values between compound treated and control (without compound) samples.

## Supporting Information

Figure S1 **CADA dose-dependently down-modulates hCD4 fusion protein.** (A) CHO cells, stably expressing CD4-YFP (hCD4 fused at its COOH-terminus to the YFP), were treated for 24 h with a serial 1∶10 dilution of CADA. Cells were collected, stained with anti-CD4 mAb, and analyzed by flow cytometry for receptor expression by simultaneous detection of CD4 and YFP. One representative experiment is shown. (B) Cell lysates of (A) were analyzed by immunoblotting for CD4-YFP fusion protein (∼80 kDa) with anti-GFP or anti-clathrin antibodies. A non-transfected control is included (first lane). Molecular mass is in kDa. (C) CADA does not affect WT CCR5 receptor expression. Freshly isolated primary T-cells from two different donors were incubated with CADA for 3 days, stained for CCR5, and analyzed by flow cytometry for receptor expression. Bars represent the mean fluorescent intensities (MFI) for CCR5 of 10,000 analyzed cells. (D) Scheme of the CD4-CCR5 fusion protein, composed of the NH_2_-terminal part of hCD4 fused to the seven TM domain G protein coupled chemokine receptor CCR5. (E) HEK293T-cells expressing hCD4-CCR5 (construct shown in (D)) were treated for 48 h with control medium or CADA and stained with anti-CD4 or anti-CCR5 antibodies to determine surface expression levels of mutant CD4 by flow cytometry. Graph shows relative receptor expression levels of CADA-treated cells as compared to non-treated controls. (F) CADA does not affect mRNA steady state levels of hCD4. Three different cell lines (U87.CD4, CHO.CD4, and SupT1) were incubated with CADA for 24 h and subjected to real-time reverse transcription (RT)-PCR for CD4. The results were analyzed in terms of CT values. The triplicate values are shown for control and CADA-treated samples. In parallel, cell surface CD4 was determined by flow cytometry, confirming a profound down-modulation of surface CD4 with CADA (>80% reduction in CD4 protein levels).
(EPS)

Figure S2 **CADA does not generally target membrane and secreted proteins.** (A) U87 cells were pretreated with CADA (5 µM) or DMSO for 1 h before starvation for 30 min in Met/Cys free medium with CADA, DMSO, or 50 µg/ml CHX. Cells were pulsed for 30 min, washed, and incubated in fresh medium without serum for 90 min. After collection of supernatant proteins (Media) cells were lysed in NP-40 buffer to obtain total cell lysate (Lysate). Glycosylated proteins were isolated from total lysate with Concanavalin A (ConA) agarose beads (Glycosylated). (B) Same as in (A) for SupT1 cells. Where indicated, pretreated cells were activated with 2 µg/ml phytohaemagglutinin (PHA) for 1 h before they were incubated in starvation medium. The secreted protein fraction of a second experiment in which the cells responded to the same PHA-stimulus more effectively is included.
(EPS)

Figure S3 **CADA inhibits co-translational translocation of an hCD4 SP-containing control protein.** To support our findings that CADA acts solely through the SP-containing N-terminus of hCD4, we generated a cDNA encoding a non-CD4 related soluble protein with the signal sequence and seven N-terminal residues of hCD4 attached to its N-terminus. For ease of detection, YFP was selected as reporter protein. (A) Schematic representation of the YFP construct used. Numbers represent the corresponding amino acids of the pre-protein. The N-terminal SP is indicated by a black rectangle. (B) Dose-response curve showing the effect of CADA on YFP expression *in cellulo*. HEK293T cells were transiently transfected with the expression plasmid and left either untreated or treated with CADA for 48 h. YFP levels were determined by flow cytometry and normalized to non-treated controls (*n* = 5). IC_50_ value for CADA is 1.28 µM. (C) Transcripts encoding the YFP construct were translated *in vitro* in rabbit reticulocyte lysate in the presence of [^35^S]methionine and, where indicated, RMs and/or CADA (15 µM). Samples were separated by SDS-PAGE and analyzed by autoradiography. The position of precursor and signal-cleaved species is indicated. (D) HEK293T cells were transiently transfected with the expression plasmid and left either untreated or treated with CADA (10 µM). The proteasome inhibitor MG132 (5 µM) was added 5 h post-transfection and cells were incubated for another 18 h. Next, cell lysates were analyzed by immunoblotting with anti-GFP or anti-clathrin antibodies. In the right panel, the input of the CADA sample is enhanced in order to visualize the rescued precursor protein [hCD4]-(7)-YFP. (E) Schematic representation of the V5-tagged CD4 construct used in Figure 4F. Numbers represent the corresponding amino acids of the pre-protein. The N-terminal SP is indicated by a black rectangle. The simian virus 5 (V5) epitope (GKPIPNPLLGLD) was incorporated at the N-terminus of mature CD4 between residue 32 and 33. Note that the first two residues of V5 (residue G and K) were already present in CD4 at positions 31 and 32.
(EPS)

Figure S4 **CADA specifically binds to the SP of human CD4 in a dose-dependent way.** (A) Dose-dependent binding of CADA to the SP of hCD4. Peptides composed of the signal sequence and eight N-terminal residues of mature CD4, plus a PEG11 linker and a biotinylated lysine at the C-terminus, were chemically synthesized and captured on a streptavidin C1 chip for SPR analysis. The chip density was 104 resonance units (RUs). Graph shows binding to SP of hCD4 for increasing doses of CADA (µM). Insert gives corresponding RU values. (B) As in (A), but for the binding of SRP to the SP of mouse CD4. The chip density was 130 resonance units (RUs). Graph shows dose response of SRP (nM) to mouse CD4 SP. (C) Dose-dependent binding of CADA (black solid and dotted lines) to the SP of hCD4 (left panel) but not to the bovine pPL SP (right panel). The chip density was 123 RUs for hCD4 and 215 RUs for pPL. For pPL, the binding of 100 µM MFS105 was also tested (red line). (D) Repeat experiment as presented in Figure 5C. The chip density was 121 RUs. (E) Chemical structure of MFS105 (MFS), molecular weight (MW) = 466.
(EPS)

Figure S5 **CADA interacts primarily with the hydrophobic H-region of the human CD4 signal peptide.** (A) *In vitro* translocation of the human-murine chimaera from Figure 5D. Translation and translocation of the CD4 constructs were performed as described in the legend to Figure 4C and 4D. One representative gel out of two is shown. (B) Translocation data of (A) were quantified by phosphorimager analysis and the translocation efficiencies as normalized to control were calculated (mean values of two experiments). The IC_50_ values of CADA for each construct are included for the *in vitro* translocation experiments. (C) Insertion of the hydrophobic H-region of hCD4 into the SP of mouse CD4 results in sensitivity to CADA. The sequences of the SP of WT mouse CD4 and the murine-human chimaera are indicated at the top of the graph. Graph shows the flow cytometry analysis of HEK293T cells transiently transfected with the expression plasmids of WT murine CD4 and murine-human chimaera left either untreated or treated with CADA for 48 h. Cells were collected and stained for hCD4. CD4 expression levels were normalized to non-treated controls (*n* = 3). IC_50_ value for CADA = 2.45 µM. *p<0.01. (D) Insertion of the hydrophobic H-region of hCD4 into the SP of bovine pPL results in sensitivity to CADA. The sequences of the SP of WT pPL and the pPL-human chimaera are indicated at the top of the graph. Graph shows the *in vitro* translocation efficiencies as described in (A) and (B). pPL translocation levels were normalized to non-treated controls (*n*≥2). *p<0.01.
(PDF)

Figure S6 **The alpha-helix terminator Proline 20 (P20) in the SP of hCD4 is necessary but not sufficient for CADA sensitivity.** (A) Occurrence of proline residues in different SPs. Clustal W Multiple alignment of SP sequences, compared to the hCD4 SP. (B) The Clustal W Multiple alignment was used to create a weblogo (WebLogo 3.4) where the first position corresponds to M1 of hCD4. There is no dominant occurrence of proline residues at the C-terminus of the hydrophobic core region of SPs. (C) Different proline mutants were tested for CADA sensitivity. HEK293T cells were transiently transfected with the expression plasmids and left either untreated or treated with 10 µM CADA for 48 h. Cells were collected and stained for hCD4. CD4 expression levels were normalized to non-treated controls. The SP sequences of the six constructs used are listed in the same order as presented in the graph from left to right.
(EPS)

Figure S7 **CADA inhibits co-translational translocation of hCD4-pPL nascent chains.** (A) Autoradiogram of truncated *in vitro* translated [^35^S]methionine-labelled [hCD4]-(7)-pPL. Nascent chains of increasing length were synthesized in the presence of RM, with or without CADA (15 µM). Next, samples were treated with puromycin to release the nascent chains from ribosomes. Equal aliquots of the translated material were left untreated or treated with PK. Samples were separated by SDS-PAGE and analyzed by autoradiography. Intact peptidyl-tRNA bands (arrow), released nascent chains (open arrowhead), signal-cleaved species (solid arrowhead), and SPs (open circle) are indicated. Molecular mass is in kDa. Note that for the 102-mers, addition of RM already resulted in some SP-cleaved species (lane 21). (B) Fraction of translocated nascent chains (after puromycin release) in the absence or presence of CADA (as in (A)) quantified and plotted against chain length (means ± standard deviation [SD]; *n*≥3). Note that the optimal chain length for efficient SP-cleavage and C-terminal translocation is 80 residues. (C) Inhibition by CADA of the translocation of [hCD4]-(7)-pPL nascent chains from (B). The fraction of translocated nascent chains was quantified for CADA samples and compared to that of the untreated controls in order to determine the percentage translocation-inhibition for CADA (means ± SD; *n*≥3).
(EPS)

Figure S8 **Effect of CADA on the translocation of N-terminal extended N-glycosylation tagged proteins.** (A) Autoradiogram of *in vitro* translated N-tagged [hCD4]-(7)-pPL full length constructs represented in Figure 7A. Translation was as described in the legend to Figure 4D. The positions of full length precursor (open arrowhead) and signal-cleaved translocated (solid arrowhead) species are indicated. N-glycosylated species are almost non-existing because the SP (that contains the N-glycosylation site) is cleaved off from the pre-protein during translocation. (B) Deglycosylation with Endo H of N-terminal glycosylated 17+62-mers from Figure 7C. (C) CADA dose-dependently inhibits both the N-terminal glycosylation and the C-terminal translocation with SP-cleavage of the 17+80-mers. Same as in Figure 7B and 7C, except that a 1∶10 serial dilution of CADA was used. (D) Graph shows translocation data of (C) quantified by phosphorimager analysis. (E) Intact nascent chains (NC-tRNA) from Figure 7B were exposed to PK in the absence or presence of 0.5 M NaCl. Note that only the control samples of the 17+71-mers were high-salt resistant, whereas in the presence of CADA these nascent chains became high-salt sensitive.
(EPS)

Figure S9 **N-terminal N-glycosylation is dependent on extension length.** (A) Extension of the SP N-terminus requires minimum 17 residues for efficient N-terminal glycosylation. Schematic representation of the different constructs with a diagnostic glycosylation site at the N-terminus of the SP. Construct N8 is a G4S mutant of WT hCD4 SP with a NRS glycosylation site. The other constructs contained an N-terminal extension of the SP. The residues of the SP of hCD4 are in black, whereas those of the inserted tag are depicted in grey. The glycosylation sites are in italic and underlined. The numbering (N8, N13, N18, and N24) refers to the position of the asparagine residue of the glycosylation site, relative to the N-terminal end of the H-domain of the SP (leucine residue, L10 of WT hCD4 SP). (B) Autoradiogram of truncated *in vitro* translated [^35^S]methionine-labelled [hCD4]-(7)-pPL RNCs in the presence of RM. All constructs were C-terminal truncated at the same residue, i.e., residue 33 of the mature protein (resulting in the 58-mer for WT [hCD4]-(7)-pPL). Intact RNCs (NC-tRNA, arrow) and N-terminal glycosylated RNCs (asterisk) are indicated. (C) Fraction of N-terminal glycosylated nascent chains (after puromycin release) for the constructs from (A). Fractions are quantified and plotted against chain length. Here, x represents the variable length of the N-terminal extension, with x = 0 for N8, x = 7 for N13, x = 10 for N18, and x = 17 for N24. (D) Same as in (C), but for the fraction of C-terminal translocated (with SP-cleavage) nascent chains. Note that for all constructs optimal chain length for efficient C-terminal translocation is x+80 residues. (E) CADA does not inhibit signal peptidase activity. Signal peptidase was extracted from microsomes and used for *in vitro* cleavage of leader sequences from control bovine pre-prolactin (pPL, right panel) and [hCD4]-(7)-pPL (CD4, left panel) in the absence or presence of CADA (15 µM). The positions of full length precursor (open arrowhead) and signal-cleaved (solid arrowhead) species are indicated. In lane 2, intact membranes (RM) were added to the translation mixture to determine normal SP cleavage. In lanes 3–5, full length protein was first translated *in vitro* and denatured in SDS buffer before administration of the extracted signal peptidase (lanes 4 and 5).
(EPS)

Data S1 Data for graphs in Figures 1-7 and Figures S1-S9.
(XLSX)

## Abbreviations

CADA: cyclotriazadisulfonamide
CHX: cycloheximide
ER: endoplasmic reticulum
hCD4: human CD4
mCD4: mouse CD4
PK: proteinase K
pPL: pre-prolactin
PTC: peptidyltransferase centre
RM: rough microsome
RNC: ribosome-nascent chain complex
SA: signal anchor
SP: signal peptide
SPR: surface plasmon resonance
SRP: signal recognition particle
TM: transmembrane
WT: wild-type
YFP: yellow fluorescent protein
