# Supplementary figures and images for: Signal Peptide-Binding Drug as a Selective Inhibitor of Co-Translational Protein Translocation
Source: PLoS Biol. 2014 Dec 2;12(12):e1002011. doi: 10.1371/journal.pbio.1002011 (PMC4251836; doi:10.1371/journal.pbio.1002011)

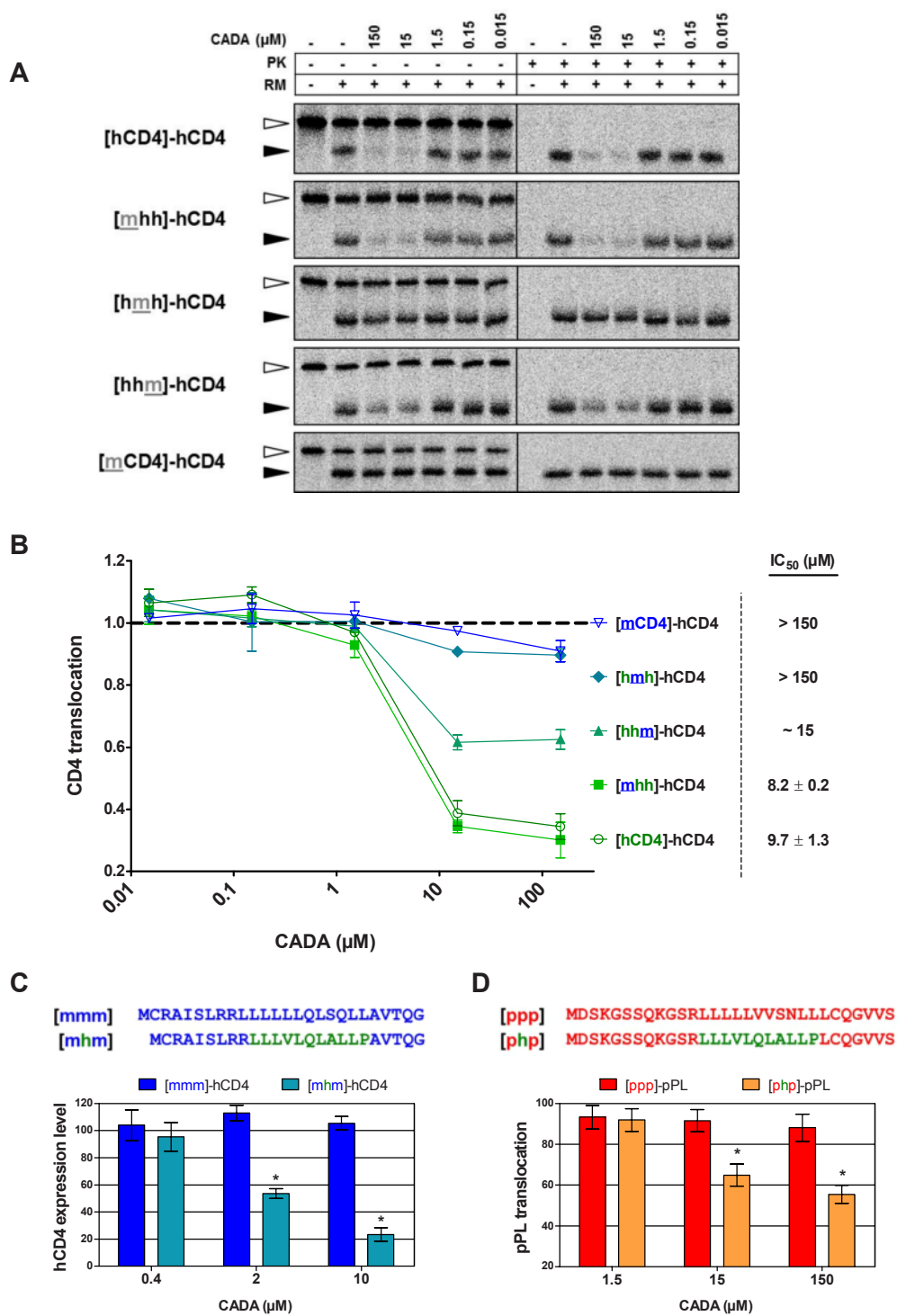

Supplement: Figure S5 — CADA interacts primarily with the hydrophobic H-region of the human CD4 signal peptide. (A) In vitro translocation of the human-murine chimaera from Figure 5D. Translation and translocation of the CD4 constructs were performed as described in the legend to Figure 4C and 4D. One representative gel out of two is shown. (B) Translocation data of (A) were quantified by phosphorimager analysis and the translocation efficiencies as normalized to control were calculated (mean values of two experiments). The IC50 values of CADA for each construct are included for the in vitro translocation experiments. (C) Insertion of the hydrophobic H-region of hCD4 into the SP of mouse CD4 results in sensitivity to CADA. The sequences of the SP of WT mouse CD4 and the murine-human chimaera are indicated at the top of the graph. Graph shows the flow cytometry analysis of HEK293T cells transiently transfected with the expression plasmids of WT murine CD4 and murine-human chimaera left either untreated or treated with CADA for 48 h. Cells were collected and stained for hCD4. CD4 expression levels were normalized to non-treated controls (n = 3). IC50 value for CADA = 2.45 µM. *p<0.01. (D) Insertion of the hydrophobic H-region of hCD4 into the SP of bovine pPL results in sensitivity to CADA. The sequences of the SP of WT pPL and the pPL-human chimaera are indicated at the top of the graph. Graph shows the in vitro translocation efficiencies as described in (A) and (B). pPL translocation levels were normalized to non-treated controls (n≥2). *p<0.01. (PDF) [file pbio.1002011.s005.pdf]
